# A total pleural covering of absorbable cellulose mesh prevents pneumothorax recurrence in patients with Birt-Hogg-Dubé syndrome

**DOI:** 10.1186/s13023-018-0790-x

**Published:** 2018-05-15

**Authors:** Teruaki Mizobuchi, Masatoshi Kurihara, Hiroki Ebana, Sumitaka Yamanaka, Hideyuki Kataoka, Shouichi Okamoto, Etsuko Kobayashi, Toshio Kumasaka, Kuniaki Seyama

**Affiliations:** 1 Pneumothorax Research Center and Department of General Thoracic Surgery, Tamagawa Hospital, Nissan Institute of Medicine, 4-8-1 Seta, Setagaya-Ku, Tokyo, 158-0095 Japan; 2 Departments of General Thoracic Surgery, Departments of General Thoracic Surgery, Graduate School of Medicine, Chiba University, Chiba, Japan; 3 Division of Respiratory Medicine, Juntendo University Faculty of Medicine and Graduate School of Medicine, Tokyo, Japan; 4 Department of Pathology, Japanese Red Cross Medical Center, Tokyo, Japan; 5 The Study Group for Pneumothorax and Cystic Lung Diseases, Tokyo, Japan

**Keywords:** Birt-Hogg-Dubé syndrome, Pneumothorax, Pleural covering, Multiple cystic lung disease

## Abstract

**Background:**

Birt-Hogg-Dubé syndrome (BHDS) is a recently recognized inherited multiple cystic lung disease causing recurrent pneumothoraces. Similarly to the lesions in patients with lymphangioleiomyomatosis (LAM), the pulmonary cysts are innumerable and widely dispersed and cannot all be removed. We recently described a total pleural covering (TPC) that covers the entire visceral pleura with oxidized regenerated cellulose (ORC) mesh. TPC successfully prevented the recurrence of pneumothorax in LAM patients. The purpose of this study was to evaluate the effect of an ORC pleural covering on pneumothorax recurrence in BHDS patients.

**Results:**

This retrospective study enrolled a total of 81 pneumothorax patients with the diagnosis of BHDS who underwent 90 covering surgeries from January 2010 to August 2017 at Tamagawa Hospital. During the first half of the study period, a lower pleural covering (LPC) which covered the affected area with ORC mesh was mainly used to treat 38 pneumothoraces. During the second half of the study period, TPC was primarily performed for 52 pneumothoraces. All the thoracoscopic surgeries were successfully performed without serious complications (≥ Clavien-Dindo grade III). The median follow-up periods after LPC/TPC were 66/34 months, respectively. Pneumothorax recurrence rates after LPC at 2.5/5/7.5 years postoperatively were 5.4/12/42%, respectively; none of the patients who had underwent TPC developed postoperative pneumothorax recurrence (*P* = 0.032).

**Conclusions:**

TPC might be an effective option for surgical treatment of intractable pneumothorax in patients with BHDS.

**Electronic supplementary material:**

The online version of this article (10.1186/s13023-018-0790-x) contains supplementary material, which is available to authorized users.

## Background

Birt-Hogg-Dubé syndrome (BHDS), a rare, inherited autosomal dominant genodermatosis caused by a germline mutation in the folliculin (*FLCN*) gene, was first reported in 1975 and 1977 [1, 2]. The three major manifestations of BHDS are fibrofolliculomas and trichodiscomas of the skin, renal tumors, and multiple lung cysts [3]. These numerous lung cysts, which are predominantly located in the middle to lower lung fields, lateral to the mediastinum, and in the interlobar area, have thin walls, round-to-oval shapes, varying sizes, and often abut peripheral pulmonary vessels [4, 5]. These peculiar features of pulmonary cysts in BHDS are impossible to treat by standard surgical methods for pneumothorax, which include resection and/or ligation of all identifiable bullae. Accordingly, repeated pneumothoraces in patients with BHDS tend to be intractable [6].

The American College of Chest Physicians (ACCP) and British Thoracic Society (BTS) guidelines for treatment of pneumothorax recommend additional procedures for spontaneous pneumothorax to minimize postoperative recurrence. These procedures include abrasion of the parietal pleura or pleurectomy after bullectomy [7, 8]. However, pleurodesis resulting from pleural abrasion or pleurectomy may disturb normal pleural physiology and lead to difficulties in future surgical procedures [9].

We reported that partial pleural covering by ORC mesh for the additional treatment of spontaneous pneumothorax after bullectomy reduced postoperative recurrence [10]. Furthermore, Lee and colleagues performed a prospective randomized, large-scale clinical trial and reported successful results with the use of partial covering by ORC mesh around the staple lines after bullectomy for patients with primary spontaneous pneumothorax [11]. Pleural covering by ORC mesh might replace mechanical pleurodesis.

The rare multiple cystic lung diseases, which include lymphangioleiomyomatosis (LAM), BHDS, cystic fibrosis, Ehlers-Danlos syndrome (type IV), Marfan syndrome, Langerhans cell histiocytosis, amyloidosis, Sjögren syndrome, and lymphocytic interstitial pneumonitis, can cause repeated pneumothoraces. For instance, LAM patients were reported to develop frequent intractable pneumothoraces because of multiple and widely dispersed fragile pulmonary cysts [9]. We and other investigators recently reported that total covering (TPC) by ORC mesh successfully prevented the recurrence of pneumothorax in LAM patients [12, 13]. To treat intractable pneumothorax in patients with BHDS, we first used an ORC mesh for lower pleural covering (LPC), which covered the areas impacted by the lesions (For details see Additional file 1: Figure S1). However, after observing the favorable effects of an ORC mesh for TPC of the fragile lungs of LAM patients, and being aware of the small intangible bullae in the upper lung fields of BHDS patients, we gradually changed our approach to the treatment of pneumothorax for patients with BHDS from ORC-mesh LPC to ORC-mesh TPC. Here, we report the results of our study that was aimed at determining and comparing the clinical outcomes of BHDS patients who either underwent LPC or TPC surgery.

## Methods

### Patients

The medical records of BHDS patients who underwent video-assisted thoracoscopic surgery (VATS) covering procedures using ORC mesh for pneumothorax at Nissan Tamagawa Hospital between January 2010 and August 2017 were retrospectively analyzed. The procedures treated a total of 90 consecutive pneumothoraces in 81 BHDS patients, nine of whom underwent bilateral lung surgeries. The pleural covering procedure using ORC mesh was performed to avoid performing pleurodesis and/or repeated surgeries for BHDS patients whose recurrent episodes of pneumothorax were not likely to be controlled by conventional treatment modalities. Accordingly, the efficacies of LPC, which was confined to the visibly affected area (includes the middle to lower lung field), and TPC, which was used for the entire visceral pleura of the affected unilateral lung, were retrospectively analyzed.

The diagnosis of BHDS was established according to the diagnostic criteria of the European BHD consortium [14], and genetic testing of *FLCN* was performed by a previously described method [15]. The following types of data were collected: age of patient when TPC or LPC was performed; surgical data such as number of ports used for thoracoscopic surgery, number of ORC meshes, amount of fibrin sealant, operating time, and surgical complications; number of pneumothorax recurrence(s) after the covering surgery; observation period; frequency of pneumothorax before and after the surgery; and postoperative complications, which were defined and graded according to the Clavien-Dindo classification of surgical complications [16]. This retrospective study was approved by the ethics committee of our institution (IRB No. TAMA2015005).

### Distribution of bullae in patients with BHDS, and procedure performed (lower or total pleural covering)

**Fig. 1:**
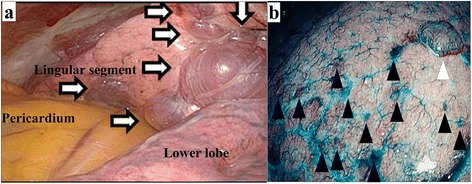
Thoracoscopic findings of multiple lung cysts in two representative patients of Birt-Hogg-Dubé syndrome: **a** shows a thoracoscopic finding of the unique distribution of multiple lung cysts adjacent to the pericardium and the interlobar region (white arrows in a representative case). **b** is a thoracoscopic narrow-band image emphasizing a protuberant bulla (white arrowhead) and multiple flat cysts around interlobular septa (black arrowheads in a representative case)

The lung cysts of patients with BHDS are predominantly located in the middle to lower lung fields (Fig. 1a: white arrows in a representative case), LPC was firstly used for BHDS-affected lungs, and the covering approximately corresponded to the middle-to-lower lung fields. Thanks to advances in high-definition thoracoscopy (OLYMPUS LTF-S190-10 surgical videoscope with VISERA ELITE OTV-S190® video processor and OLYMPUS CLV-S190 light source; Olympus, Tokyo, Japan), narrow-band-imaging (NBI) modes (filtered xenon light with emission bands at 415 nm and at 540 nm) revealed not only protuberant cysts (Fig. 1b: a white arrowhead in a representative case), but also flat and small bullae over the entire visceral pleura contiguous to the interlobular septa (Fig. 1b: black arrowheads in a representative case), which were difficult to detect by standard white-light imaging (unfiltered xenon light). The TPC procedure was described previously [12] for fragile LAM lung cysts. Briefly, TPC consisted of totally enclosing the entire surface of BHDS lungs on the surgical side by approximately 14 sheets of ORC mesh (Ethicon SURGICEL absorbable Hemostat gauze; Johnson & Johnson, Brunswick, NJ, USA), followed by drops of fibrin glue (Bolheal; Chemo-Sero-Therapeutic Research Institute (Kaketsuken), Kumamoto, Japan) (Additional file 2: Figure S2 and Additional file 3: Video S1). To complete the TPC procedure, a 20-Fr drainage tube was placed into the apex of the thoracic cavity. Scrutiny confirmed that the ORC-covered lungs were fully expanded (Additional file 3: Video S1).

### Follow up after lower or total pleural covering

The first follow-up examination was performed 2 weeks after each patient was discharged from the hospital, and included a physical examination and a chest x-ray. Subsequent follow up was performed every 3 to 4 months. For patients who developed clinical signs and symptoms of pneumothorax, a prompt medical check-up was mandatory. If a recurrence was suspected, computed tomography (CT) was performed for confirmation.

#### Statistical analysis

Statistical analysis was performed using the StatView (version 4.5) software package (Abacus Concepts, Berkeley, CA, USA), according to the statistical and data reporting guidelines for the European Journal of Cardio-Thoracic Surgery and the Interactive Cardio-Vascular and Thoracic Surgery [17]. All continuous values were expressed as means ± standard deviation. The data were evaluated using the Student *t* test for comparison of continuous variables and χ^2^ test for comparison of frequencies. The recurrence probability in the surgical lung after TPC or LPC for pneumothorax was estimated by Kaplan-Meier analysis [18]; the recurrence probabilities in surgical lungs after TPC and after LPC were compared by the log-rank test. A *P*-value less than 0.05 was considered statistically significant.

## Results

### Patient characteristics (Table 1)

Characteristics of the 81 BHDS patients in this study are summarized in Table 1. All the patients were Asians who had repeated pneumothoraces and the diagnosis of BHDS was established by *FLCN* genetic testing [15]. The median age (range) at the covering surgery was 40 years (22–68 years). Seventy-two of 81 BHDS patients underwent unilateral pleural covering surgeries consisting of 44 TPCs and 28 LPCs; nine BHDS patients underwent bilateral pleural covering, which consisted of three bilateral TPCs; four bilateral LPCs; and two combinations of TPCs on the left and LPCs on the right.

**Table 1:** Characteristics of the study population (*n* = 81)

Gender (male/female)	46/35
Age at the surgery - median (range)	40 yrs. (22–68)^a^
Surgical procedures - total	90
TPC only on the right side	26
TPC only on the left side	18
TPC bilaterally	6
LPC only on the right side	11
LPC only on the left side	17
LPC bilaterally	8
TPC on the left and LPC on the right	4

*TPC* total pleural covering, *LPC* lower pleural covering. ^a^Since seven patients underwent TPC or LPC bilaterally, and two patients underwent TPC on the left and LPC on the right, a total of 90 surgeries were used for calculations

### Operative data (Table 2)

TPCs were performed entirely under VATS using a mean of 4.0 ± 0.28 (range: 3–5) ports. The mean operative time for TPC was 140 ± 35 (range: 76–245) minutes. The mean number of ORC mesh sheets (10.2 cm × 20.3 cm) used for TPC was 14 ± 2.3 (range: 7–18) sheets per patient, with a mean volume of 9.1 ± 2.5 (range: 3–15) mL of fibrin glue. LPCs were performed entirely under VATS using a mean of 3.3 ± 0.45 (range: 3–4) ports. The mean operative time for LPC was 110 ± 32 (range: 59–186) minutes. The mean number of ORC mesh sheets (10.2 cm × 20.3 cm) used for LPC was 9.0 ± 2.3 (range: 5–13) sheets per patient, with a mean volume of 7.1 ± 1.9 (range: 3–10) mL of fibrin glue. No severe complications (≥ Clavien-Dindo grade III) were recorded during the TPCs or LPCs.

**Table 2:** Patient characteristics stratified by TPC or PPC

	TPC 52	LPC 38	*P* value
Backgrounds:			
Age at the first pneumothorax episode (years)	38 ± 9.5^a^	38 ± 11^a^	0.944
Age at surgery	40 ± 9.9^a^	42 ± 9.7^a^	0.431
Gender (male/female)	28 / 24	18 / 20	0.544
Laterality (right/left)	29 / 23	21 / 17	0.962
Surgical approach:			
VATS / Open thoracotomy	52 / 0	38 / 0	N/A
Number of used ports	4.0 ± 0.28^a^	3.3 ± 0.45^a^	< 0.001
Surgical material:			
ORC mesh (10.2 × 20.3 cm)	14 ± 2.3^a^	9.0 ± 2.3^a^	< 0.001
Amount of fibrin sealant (mL)	9.1 ± 2.7^a^	7.1 ± 1.9^a^	< 0.001
Operating time (minutes)	140 ± 35^a^	110 ± 32^a^	< 0.001
Severe complication (≥ grade IIIa)^b^ (during surgery)	0	0	N/A
Follow-up period after surgery (months)	38 ± 22^a^	65 ± 20^a^	< 0.001

*TPC* total pleural covering, *LPC* lower pleural covering, *VATS* video-assisted thoracoscopic surgery, *ORC* oxidized regenerated cellulose, *N/A* not applicable

^a^Mean ± standard deviation, ^b^Clavien-Dindo classification

### Postoperative complications and postoperative course of patients undergoing total or lower pleural covering

TPC: Postoperative complications greater than Clavien-Dindo grade III after TPC were found in 2 of 52 procedures (3.8%). The two complications were each a grade IIIa complication that required reinsertion of a chest tube for delayed occurrence of an air leak, using local anesthesia. For the entire group of TPC patients, chest drainage tubes were removed at a mean of 7.7 ± 4.6 (range: 4–30) days after surgery, and patients were discharged from the hospital at a mean of 9.5 ± 4.8 (range: 5–32) days after surgery.

LPC: postoperative complications greater than Clavien-Dindo grade III after LPC were found in 3 of 38 procedures (7.9%). Two complications were a grade IIIa complication that required reinsertion of a chest tube using local anesthesia for delayed occurrence of an air leak and 1 grade IIIb complication that consisted of localized empyema in the chest cavity requiring curettage under general anesthesia. For the entire group of LPC patients, chest drainage tubes were removed at a mean of 7.0 ± 4.1 (range: 3–20) days after surgery, and patients were discharged from the hospital at a mean of 8.5 ± 4.1 (range: 5–21) days after surgery.

### Recurrence rate after pleural covering surgery for pneumothorax

**Fig. 2:**
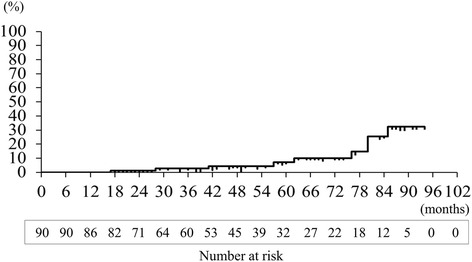
Overall recurrence rate of pneumothorax after pleural covering surgery for patients with Birt-Hogg-Dubé syndrome: Kaplan-Meier graph estimating the recurrence probability after a total of 90 covering surgeries to prevent pneumothorax, as follows: 2.7% at 2.5 years, 7.1% at 5.0 years, and 32% at 7.5 years, with a median postoperative follow-up period of 48 (range: 6.7–94) months

**Fig. 3:**
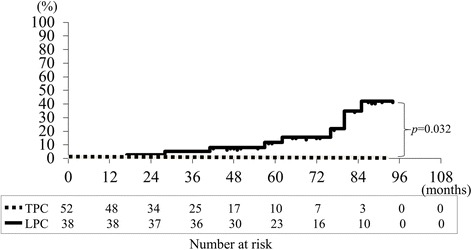
Comparing Total Pleural Covering (TPC) with Lower Pleural Covering (LPC): recurrence rate after pneumothorax surgery. Kaplan-Meier estimates of the recurrence probability after 52 TPCs to prevent pneumothorax, as follows; 0% at 2.5, 5.0 , and 7.5 years after surgery (dotted line). The recurrence probability after 38 LPCs to prevent pneumothorax, as follows: 5.4% at 2.5 years; 12% at 5.0 years; and 42% at 7.5 years after surgery (solid black line). The rog-rank test shows that TPC is superior to LPC (*P* = 0.032)

Kaplan-Meier analysis of a median follow-up period of 48 (range: 6.7–94) months showed the following recurrence rates after all 90 surgeries for pneumothorax in 81 patients with BHDS: 2.7% at 2.5 years, 7.1% at 5.0 years, and 32% at 7.5 years (Fig. 2). Since the postoperative recurrence rate gradually increased each year, a subset analysis comparing the surgical outcomes of patients undergoing LPC or TPC was performed. The postoperative recurrence probability on the surgical side after LPC for pneumothorax, as estimated by Kaplan-Meier analysis, was 5.4% at 2.5 years; 12% at 5.0 years, and 42% at 7.5 years (Fig. 3). Compared to LPC, BHDS patients after TPC showed significantly better results, with no recurrence of pneumothorax on the surgical side (Fig. 3; *P* = 0.032).

### Frequency of pneumothorax episodes before and after the pleural covering surgery

**Fig. 4:**
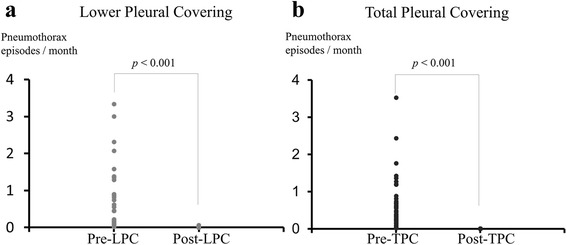
Frequency of pneumothorax episodes before and after the pleural covering surgery. **a** Comparison of the frequencies of pneumothorax episodes before and after Lower Pleural Covering (LPC): The number of pneumothorax episodes was divided by the observation period (number of months from the first pneumothorax episode to LPC or number of months after LPC). The frequency of pneumothorax (episodes/month) was significantly reduced after LPC (*P* <  0.001, Student *t* test). **b** Comparison of the frequencies of pneumothorax episodes before and after Total Pleural Covering (TPC): The number of pneumothorax episodes was divided by the observation period (number of months from the first pneumothorax episode to TPC or those after TPC). The frequency of pneumothorax (episodes/month) was significantly reduced after TPC (*P* <  0.001, Student *t* test)

The frequency of pneumothorax episodes was evaluated before and after pleural covering surgery. The frequency was significantly decreased after both LPC and TPC (Fig. 4a and b, respectively). The frequency of pneumothorax episodes on the surgical side per month before LPC was 0.629 ± 0.840 during a median observation period of 17.7 (range: 0.433–193) months. After LPC, the frequency of pneumothorax episodes per month on the surgical side was significantly lower at 0.0053 ± 0.012 during a median observation period of 66 (range: 17–94) months (Fig. 4a, *P* <  0.001). Similarly, the frequency of pneumothorax episodes on the surgical side per month before TPC was 0.555 ± 0.642 during a median observation period of 5.9 (range: 0.567–217) months. After TPC, the frequency of pneumothorax episodes on the surgical side per month was significantly reduced to zero during a median observation period of 34 (range: 6.7–93) months (Fig. 4b, *P* <  0.001).

## Discussion

The results of our retrospective analysis clearly demonstrate that the pleural covering procedure is an effective method for preventing the recurrence of pneumothorax without severe perioperative complications in patients with BHDS, and that TPC is superior to LPC. LPC covered all the visible cysts on the surgical side, to encompass approximately two thirds of the entire visceral pleura, which covers mainly the middle to lower lung field; however, LPC could not prevent pneumothorax recurring several years after surgery. TPC on the other hand, completely prevented recurrence of pneumothorax in this study, and might provide BHDS patients with freedom from the postoperative recurrence of pneumothorax.

A recent large randomized control study of an alternative treatment for primary spontaneous pneumothorax by Lee and colleagues found that bullectomy plus pleural covering on the staple line using ORC mesh and fibrin glue showed surgical outcomes comparable to those after standard surgery, which includes surgical pleurodesis. Regarding the effectiveness of an ORC covering of the pleura as opposed to pleurodesis, we confirmed in both an animal model (beagle dogs) and a clinical study that ORC mesh is the preferred material for inducing increased thickening of the visceral pleura without inducing severe visceral-to-parietal pleural adhesions [10, 12]. We recently validated that ORC mesh induces pleural thickening. We performed in vitro experiments that suggested that a mesothelial-mesenchymal transition might be a mechanism for ORC-induced pleural thickening [19]. The covering technique might eventually supersede surgical pleurodesis.

There are two types of surgical sheets available as commercial products for surgery that are composed of oxidized regenerated cellulose, as follows: 1) GYNECARE INTERCEED Absorbable Adhesion Barrier (Johnson & Johnson, Brunswick, NJ, USA) and 2) SURGICEL Original Absorbable Hemostat. The former is indicated as an adjunct to gynecologic pelvic surgery for reducing the incidence of postoperative pelvic adhesions. On the other hand, the latter product, which is composed of the same material, is used adjunctively in surgical procedures to assist in the control of capillary, venous, and small artery hemorrhages. We intentionally selected SURGICEL Original Absorbable Hemostat for pleural covering surgery, because of its excellent flexibility and plasticity when applied to an uneven surface and the interlobar regions of the lungs. Since the use of SURGICEL Original Absorbable Hemostat for TPC or LPC was off label, we needed approval by the ethical committee.

We recently reported that lung fibroblasts isolated from BHDS patients showed haploinsufficiency of *FLCN*, resulting in marked diminution in fibroblast abilities to migrate, contract, and produce extracellular matrix proteins [20], which might lead to impaired tissue repair, organ fragility and multiple lung cysts in BHDS patients. Figure 1b shows an NBI image produced by high-definition thoracoscopy, which clearly reveals numerous small flat bullae in the upper lobe, which were difficult to detect by observation under normal white light. These multiple bullae that appeared in the upper lung field, which had been believed to be a rare site for BHDS bullae from the radiological studies, could only be treated by TPC. The findings shown in Fig. 1b, which were similarly detected in 14 upper lobes in consecutive 18 BHDS patients who underwent TPC from April 2016 to July 2017 in this cohort, suggest that the entire lung is fragile, and explains the superiority of TPC to LPC in BHDS patients. Since numerous small flat bullae in BHDS patients are fragile and readily ruptured by surgical manipulations such as a grip on the lungs, we strongly recommend a careful and gentle touch during TPC.

The design of this study has some limitations. Firstly, this project was a retrospective observational analysis over an approximately 7-year period and included a small population of patients with BHDS. This study preferably should be performed as a prospective investigation that randomly allocates patients to undergo TPC or LPC; however, considering the rarity of BHDS the 52 TPC and 38 LPC procedures for 81 BHDS patients from a single hospital is a large enough number to warrant attention. Secondly, median follow up period after TPC was significantly shorter than that after LPC. Thirdly, the acceptance of TPC for BHDS patients at other hospitals might be limited by the ethical difficulties concerning the off-label use of ORC mesh for pleural covering. The use of ORC mesh for TPC needs approval by the ethical committee of each institution where it is used. However, TPC can feasibly be performed safely by any general thoracic surgeon who is experienced with VATS. We hope that the attached supplementary video file that contains the important steps in performing TPC will provide understanding, assist as an instructional instrument, and popularize the TPC technique.

## Conclusion

We used a covering procedure for patients with BHDS-affected lungs which consisted of sheets of ORC mesh to reinforce the fragile visceral pleura. The surgical outcomes of patients showed that pneumothorax recurrence on the surgical side did not develop after TPC by ORC mesh, although recurrence developed after LPC. Our results strongly support the option of TPC by ORC mesh as an effective method for preventing the recurrence of pneumothorax without significant complications. For this procedure to become mainstream of the treatment, a future study may need to be conducted in a randomized manner comparing outcomes from traditional surgical pleurodesis with TPC.

## Additional files

Additional file 1: **Figure S1.** Procedure for lower pleural covering (LPC) of right lungs: LPC covers all the visible cysts mainly in the middle to lower lung field, which encloses the surface of the 1) posterior mediastinal side of the lower lobe, 2) anterior mediastinal side of the middle lobe, 3) basal area in the lower lobe, 4) lateral side of the middle and lower lobes, and 5) interlobar surface of the lungs. (TIF 1359 kb)

Additional file 2: **Figure S2.** Technical procedure for total pleural covering (TPC) of right lungs: Schemata depicting systematic covering of an entire visceral pleura by oxidized regenerated cellulose (ORC) mesh, which encloses the surface of the 1) upper lobe, 2) basal area in the lower lobe, 3) posterior mediastinal side of the upper and lower lobes, 4) anterior mediastinal side of the upper and middle lobes, 5) lateral side of the middle and lower lobes, and 6) interlobar surface of the lungs. (TIF 1440 kb)

Additional file 3: **Video S1.** Total Pleural Covering (TPC) procedure of the right lungs in a Birt-Hogg-Dubé syndrome patient: With approximately 50% inflation of the lung, TPC using sheets of oxidized regenerated cellulose (ORC) mesh encompassed the entire surface of the lung. In detail, we covered the 1) upper lobe, 2) basal area in the lower lobe, 3) posterior mediastinal lung surface, 4) anterior mediastinal lung surfaces, 5) lateral lung surface, and 6) interlobar lung surface. (MPG 50006 kb)

## Abbreviations

ACCP: American College of Chest Physicians
BHDS: Birt-Hogg-Dubé syndrome
BTS: British Thoracic Society
FCLN: Folliculin
LAM: Lymphangioleiomyomatosis
LPC: Lower pleural covering
ORC: Oxidized regenerated cellulose
TPC: Total pleural covering
VATS: Video-assisted thoracic surgery
