# Supplementary figures and images for: A total pleural covering of absorbable cellulose mesh prevents pneumothorax recurrence in patients with Birt-Hogg-Dubé syndrome
Source: Orphanet J Rare Dis. 2018 May 15;13:78. doi: 10.1186/s13023-018-0790-x (PMC5952889; doi:10.1186/s13023-018-0790-x)

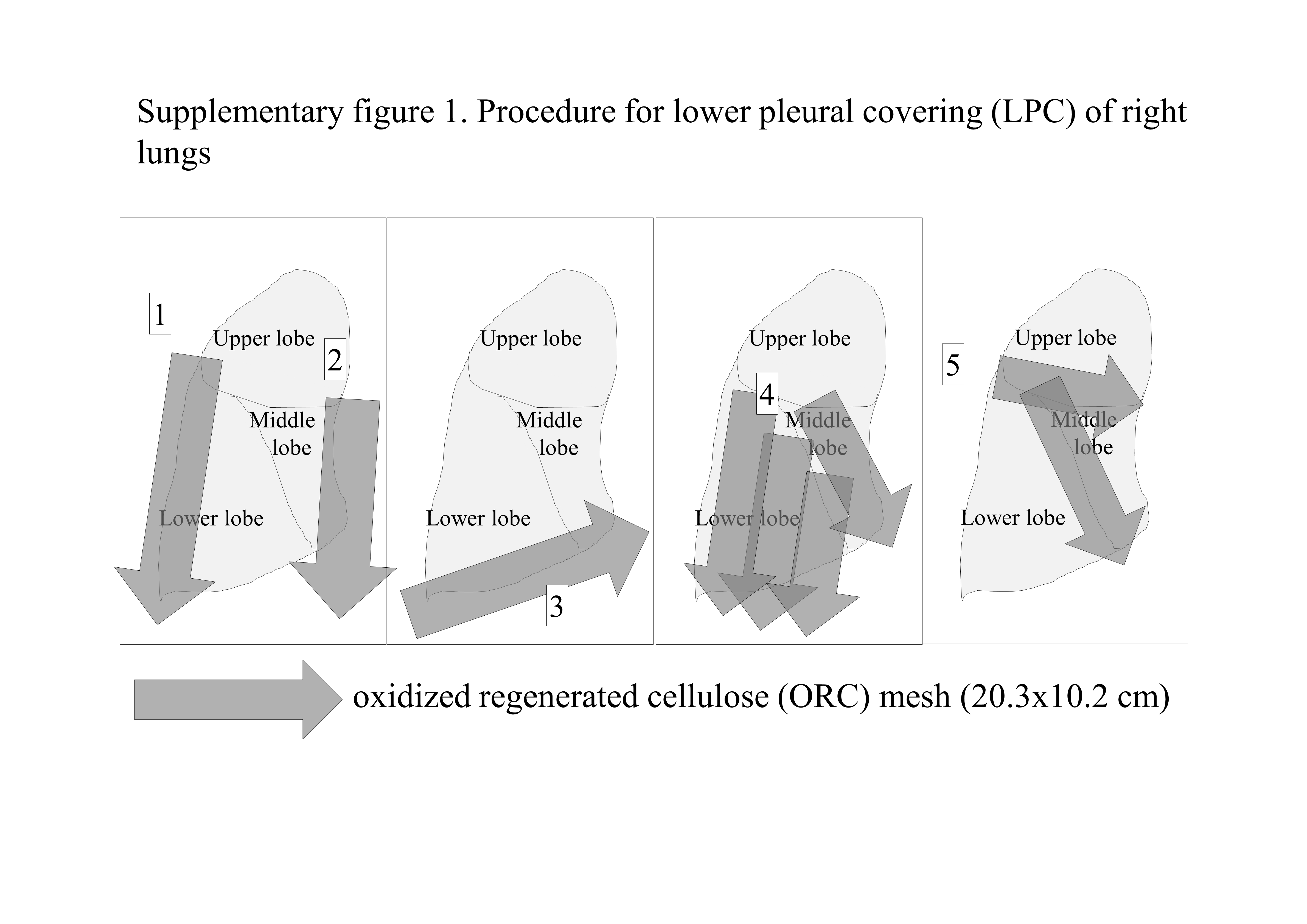

Supplement: Supplementary file 1 — Figure S1. Procedure for lower pleural covering (LPC) of right lungs: LPC covers all the visible cysts mainly in the middle to lower lung field, which encloses the surface of the 1) posterior mediastinal side of the lower lobe, 2) anterior mediastinal side of the middle lobe, 3) basal area in the lower lobe, 4) lateral side of the middle and lower lobes, and 5) interlobar surface of the lungs. (TIF 1359 kb) [file 13023_2018_790_MOESM1_ESM.tif]

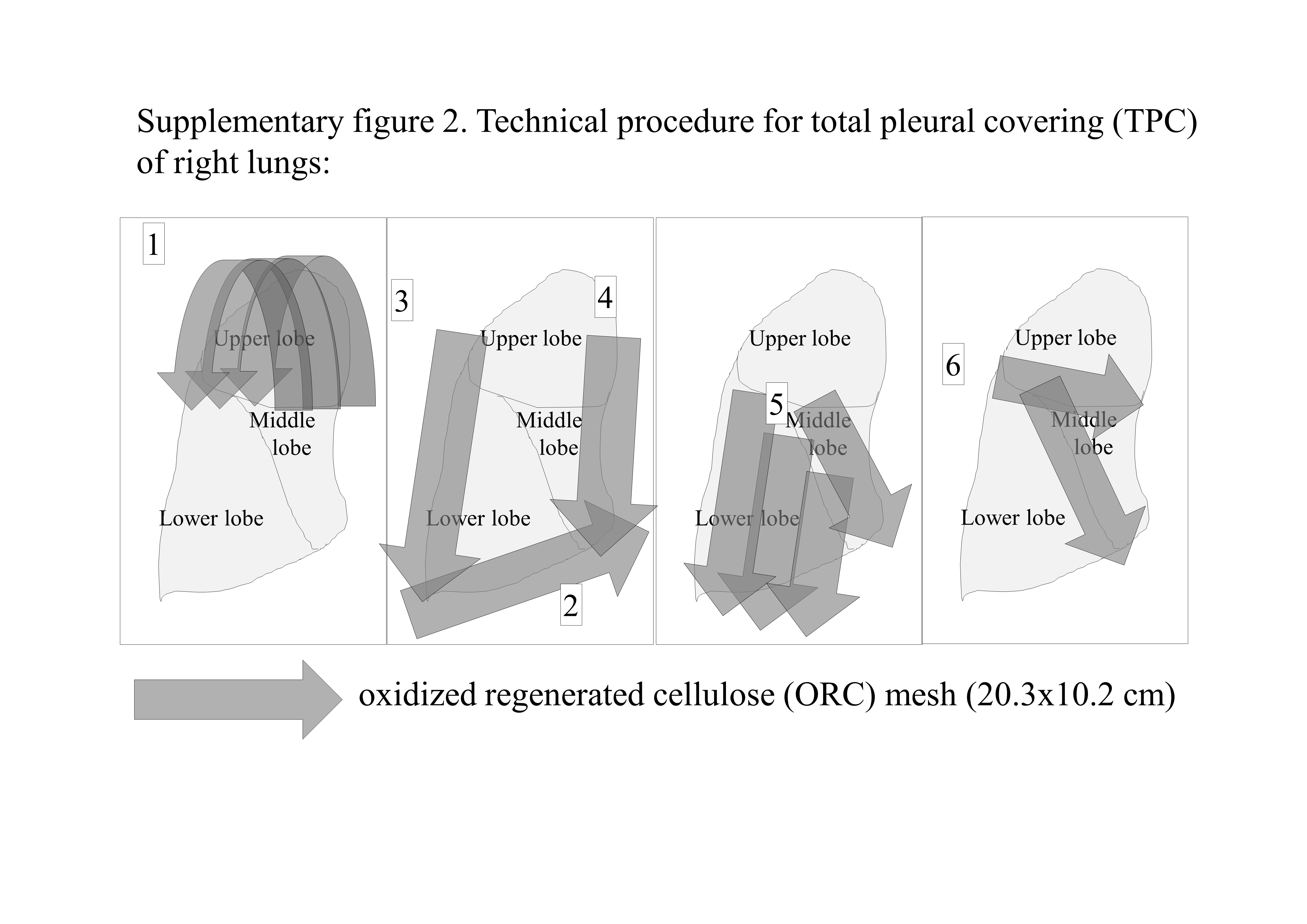

Supplement: Supplementary file 2 — Figure S2. Technical procedure for total pleural covering (TPC) of right lungs: Schemata depicting systematic covering of an entire visceral pleura by oxidized regenerated cellulose (ORC) mesh, which encloses the surface of the 1) upper lobe, 2) basal area in the lower lobe, 3) posterior mediastinal side of the upper and lower lobes, 4) anterior mediastinal side of the upper and middle lobes, 5) lateral side of the middle and lower lobes, and 6) interlobar surface of the lungs. (TIF 1440 kb) [file 13023_2018_790_MOESM2_ESM.tif]
